# Cardiac magnetic resonance of targeted annexin-iron oxide labeling detects cardiac cell death *in vivo* after doxorubicin and myocardial infarction

**DOI:** 10.1186/1532-429X-11-S1-O8

**Published:** 2009-01-28

**Authors:** Rajesh Dash, Trevor Chan, Mayumi Yamada, Marietta Paningbatan, Bat-Erdene Myagmar, Philip Swigart, Paul C Simpson, Phillip C Yang

**Affiliations:** 1 San Francisco Veterans Affairs Hospital, San Francisco, CA USA; 2 Stanford University, Stanford, CA USA

**Keywords:** Cardiac Magnetic Resonance, Cardiac Magnetic Resonance Imaging, Cardiac Cell, Signal Distribution, Superparamagnetic Iron Oxide

## Background

Heart failure from myocardial infarction (MI) or doxorubicin (DOX), used in cancer therapy, is preceded by significant cell apoptosis. Real-time, non-invasive detection of early cardiac apoptosis might impact patient treatment and outcomes. Early apoptosis is detected by Annexin V protein (ANX) binding to externalized membrane phosphatidylserine. To this end, we previously conjugated ANX to superparamagnetic iron oxide (ANX-SPIO). This conjugate specifically binds to early apoptotic cardiac cells in culture and is detectable by *in vitro* magnetic resonance imaging (MRI).

## Hypothesis

We tested whether ANX-SPIO could detect cardiac apoptosis, *in vivo*, via MRI (3 Tesla, GE Excite, WI) after ischemic or oxidative injury.

## Methods

Mice underwent LAD ligation or intraperitoneal, cardiotoxic DOX (25 mg/kg) injection. After 24–48 hours, ANX-SPIO was given by tail vein, and mice were imaged by T2-weighted cardiac MRI (3 Tesla, GE Excite).

## Results

**Figure 1:**
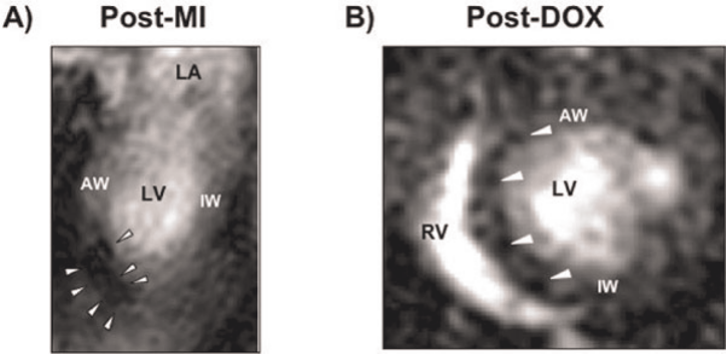
**ECG- and respiratory-gated T2-weighted cardiac MRI of mice post-MI (A, 2-chamber view) and post-DOX (B, short axis view), 30 minutes after tail vein infection of 100 μl ANX-SPIO**. Note *focal* T2 signal void (white arrows) of ANX-SPIO in antero-apex of post-MI heart, and *diffuse* septal, anterior and inferior T2 signal void in post-DOX heart (LA, left atrium; LV, left ventricle; RV, right ventricle; AW, anterior wall; IW, inferior wall).

After MI and DOX, myocardial T2 MRI signal was detectable within 30 minutes of ANX-SPIO delivery, exhibiting either a focal (MI) or diffuse (DOX) signal distribution (see Figure 1). Peak signal was evident 24 hours after ANX-SPIO delivery, and decreased over the next 2 weeks.

## Conclusion

Cardiac MRI using ANX-SPIO can accurately detect myocardial apoptosis in vivo. Distinct MRI signal distributions were noted following ischemic (MI) versus oxidative (DOX) injury. This molecular imaging strategy may help identify 'at risk' cardiac cell populations.

